# A Meta-Analysis of Array-CGH Studies Implicates Antiviral Immunity Pathways in the Development of Hepatocellular Carcinoma

**DOI:** 10.1371/journal.pone.0028404

**Published:** 2011-12-12

**Authors:** Xu Guo, Yanna, Xi Ma, Jiaze An, Yukui Shang, Qichao Huang, Hushan Yang, Zhinan Chen, Jinliang Xing

**Affiliations:** 1 State Key Laboratory of Cancer Biology, Department of Cell Biology, Cell Engineering Research Center, The Fourth Military Medical University, Xi'an, People's Republic of China; 2 Department of Clinical Immunology, Xijing Hospital, The Fourth Military Medical University, Xi'an, People's Republic of China; 3 Department of Hepatobiliary Surgery, Xijing Hospital, The Fourth Military Medical University, Xi'an, People's Republic of China; 4 Division of Population Science, Department of Medical Oncology, Kimmel Cancer Center, Thomas Jefferson University, Philadelphia, Pennsylvania, United States of America; National Cancer Institute, United States of America

## Abstract

**Background:**

The development and progression of hepatocellular carcinoma (HCC) is significantly correlated to the accumulation of genomic alterations. Array-based comparative genomic hybridization (array CGH) has been applied to a wide range of tumors including HCCs for the genome-wide high resolution screening of DNA copy number changes. However, the relevant chromosomal variations that play a central role in the development of HCC still are not fully elucidated.

**Methods:**

In present study, in order to further characterize the copy number alterations (CNAs) important to HCC development, we conducted a meta-analysis of four published independent array-CGH datasets including total 159 samples.

**Results:**

Eighty five significant gains (frequency ≥25%) were mostly mapped to five broad chromosomal regions including 1q, 6p, 8q, 17q and 20p, as well as two narrow regions 5p15.33 and 9q34.2-34.3. Eighty eight significant losses (frequency ≥25%) were most frequently present in 4q, 6q, 8p, 9p, 13q, 14q, 16q, and 17p. Significant correlations existed between chromosomal aberrations either located on the same chromosome or the different chromosomes. HCCs with different etiologies largely exhibited surprisingly similar profiles of chromosomal aberrations with only a few exceptions. Furthermore, the Kyoto Encyclopedia of Genes and Genomes (KEGG) pathway analysis indicated that the genes affected by these chromosomal aberrations were significantly enriched in 31 canonical pathways with the highest enrichment observed for antiviral immunity pathways.

**Conclusions:**

Taken together, our findings provide novel and important clues for the implications of antiviral immunity-related gene pathways in the pathogenesis and progression of HCC.

## Introduction

The development and progression of hepatocellular carcinoma (HCC) is significantly correlated to the accumulation of genomic alterations [1]. Therefore, it is important to have a clear landscape of the genomic aberrations that occur during the multistep process of hepatocarcinogenesis. Previous studies have used high-resolution molecular karyotyping analyses to provide a comprehensive catalog of structural aberrations of the whole chromosomes in HCC [2]. However, this method is highly specialized and time-consuming. As a consequence, only a very limited number of HCC cases have been evaluated in these studies. Moreover, the modest resolution of the karyotyping analysis made it difficult to fully define the overall genomic profiles of HCC in a more accurate manner. Comparative genomic hybridization (CGH) has been developed in recent years to monitor the DNA copy number changes at a global level [3]. However, traditional CGH techniques still have the limitation of modest resolution (approximately 2 Mb for amplifications and 10–20 Mb for deletions) and thus could not detect changes in smaller chromosomal regions [4]. In comparison, array-based CGH (array CGH) is a newly developed technology that allows for high-throughput and high-resolution (at 1 Mb) screening of genome-wide DNA copy number changes (either amplifications or deletions) at the gene level [5]. Array CGH combines fluorescence techniques with the microarray platform that allows for the comparison of DNA content in two differentially labeled genomes: a test genome (patient) and a reference genome (control). The microarray platform also allows for the simultaneous scanning of thousands of individual DNA sequences from the whole genome, and provides high-resolution data on the locations of identified aberrations in a single experiment. To date, array-CGH has been applied to a wide range of solid tumors, including liver, breast, gastric, kidney and bladder cancers [6], [7], [8], [9], [10]. Recently, another technology platform based on single nucleotide polymorphism (SNP) array has been developed to determine the copy number abnormalities of genomic DNA at sub-kilobase resolution [11], [12]. Except for an advantage of high resolution, this platform also has a limitation of high signal-to-noise ratio which is hard to improve[13].

Many investigators have made varying attempts to search for genes implicated in hepatocarcinogenesis. Screening for chromosomal regions with frequent gains and losses is one of the first steps toward the identification of genes. Using the traditional and array-CGH, frequent DNA copy number gains at chromosomes 1q, 8q and 20q, and frequent DNA copy number losses at 1p, 4q, 8p, 13q, 16q and 17p have been identified in HCC samples [6], [14], [15], [16], [17], [18], [19]. Some of these regions contain known candidate oncogenes or tumor suppressor genes, such as *ZNF217* (20q13) [20], *TP53* (17p13), *RB1* (13q14) [21]and *cyclin D1* (11q13) [22]. However, it is believed that the currently identified genes represented only a small percentage of causal elements in hepatocarcinogenesis and the vast majority of genes with chromosomal aberrations that may play a central role in HCC development are still unknown.

Meta-analysis is a systematic and quantitative synthesis of prior evidence [23]. It offers the opportunity to critically evaluate and statistically combine the results of comparable studies or trials in order to achieve more robust and reliable results as well as identify novel findings that are not apparent in individual studies. In previous reports, a meta-analysis of CGH data comprising of 785 HCCs has been carried out and identified significant correlations of chromosomal deletions on 4q, 13q, and 16q with hepatitis B virus (HBV) etiology [24]. Recently, using the array-CGH technology, several different studies have generated a wealth of data on more than 100 analyzed HCC samples that await a more comprehensive interpretation [16], [25], [26], [27]. The aim of this study was to identify potential genes and pathways important to HCC by utilizing the available data from published array CGH studies of human HCC.

## Materials and Methods

### Data collection of array CGH studies in HCC

Datasets for HCC array CGH studies were identified from public resources including the supplementary files of published papers, NCBI Gene Expression Omnibus (GEO, http://www.ncbi.nlm.nih.gov/geo), ArrayExpress (http://www.ebi.ac.uk) database using hepatocellular carcinoma and array-based comparative genomic hybridization as keywords. Datasets from studies using HCC cell lines were excluded. We identified four datasets with complete original data that are publicly available, including two from the supplementary files of published HCC array CGH studies and two from the GEO database (GSE8351 and GSE22635) [16], [25], [26], [27]. The detailed information was listed in Table 1. Three of the four studies used BAC clone as the hybridization probe while one used synthetic oligonucleotides as the hybridization probe. A total of 159 HCC tissue samples in these four datasets were collected, including 54 samples with HBV infection, 57 with hepatitis C virus (HCV) infection, 6 with the infections of both HBV and HCV, 3 with positive hepatitis B virus X protein (HBx), and 39 samples without viral infection.

**Table 1:** Information of 4 collected public datasets (n = 159).

ID	References	Platform	Etiology, *N*					
			All	HBV	HCV	HBV/HCV	HBx	non-viral
1	Mohini A.Patil *et al*.(2005)	BAC	44	34	3	—	—	7
2	Yasuyo Chochi *et al*.(2009)	BAC	42	6*	34*	2*	—	—
3	Christof Schlaeger *et al*.(2008)	BAC	63	11	14	4	3	31
4	Kazuya Taniguchi *et al*.(2010)	Oligo	10	3	6	—	—	1
	Total		159	54	57	6	3	39

* Information of virus infection for individual samples is unavailable. HBV, hepatitis B virus; HCV, hepatitis C virus; HBx, hepatitis B virus X protein.

### Data pre-processing for the integration across different platforms

Because the four array CGH datasets in this study were generated using different types of technical platforms that contain different numbers of probes at varied spacing and resolution, they cannot be directly compared and combined. To transform the datasets from different platforms into a common format for the purpose of meta-analysis, we pre-processed the original dataset of each study based on a procedure previously described with minor modification [24], [28]. The detailed procedure used in this study is described below:

#### First step: reconciliation of genome mapping data generated from BAC clones and oligonucleotide probes

In the original datasets, chromosomal positions of BAC and oligo probes were assigned based on the different versions of human genome assembly, such as hg15, hg17 and hg18. Therefore, we used the annotation database of UCSC human genome (hg19/GRCh37) to re-assign the start and end chromosomal positions for all BAC and oligo probes from the four datasets. The unmappable probes were excluded from further analysis.

#### Second step: assignment of chromosomal positioning anchors

Because the copy number alterations (CNAs) of chromosomal segments were detected on different scales by different probes in the four datasets, it was difficult to directly compare and integrate these datasets. To resolve this issue, we assigned a set of chromosomal positioning anchors that were composed of the start and end chromosomal positions of all the probes used in the four datasets. The log_2_-transformed DNA copy number ratio for each anchor was then determined for all the samples from different datasets based on the following principles. For any dataset, when anchors lied in the probes of the dataset, the log_2_-transformed ratio for probe was assigned to corresponding anchors. When an anchor lied outside of any detected probes of the dataset, a missing value was created and set to the anchor. Using this approach, we were able to delineate the same chromosomal positions to the different probes used in the different datasets of the four studies.

#### Third step: scaling and segmentation across multiple platforms

To integrate data across multiple sources for meta-analysis, multi-platform segmentation with proper scaling was performed by using a multi-platform circular binary segmentation (MPCBS) algorithm recently reported by Zhang et al [28], which was implemented into an R package which can be accessed on R-Forge under a project name MPCBS (http:// r-forge.r-project.org/). This algorithm, which is based on a simple multi-platform change-point model, relies on a weighted sum of t-statistics to scan for copy number changes and does not require a pre-standardization of different data sources. To minimize the computational complexity and maximize the generality, chromosomes were segmented individually and sex chromosomes were not included.

#### Fourth step: determination of gain and loss events

After segmentation, the CNAs of chromosomal segments were determined using a nonhierarchical k-means clustering (k = 3) algorithm [29]. All log_2_-transformed ratio values of the segments generated in the third step were clustered and classified into three subgroups by two threshold values (0.141 and −0.136), representing copy number gain (>0.141), copy number loss (<−0.136), and no copy number change (between −0.136 and 0.141).

#### Fifth step: evaluation of pre-processing performance

Pearson Correlation Coefficient (PCC) analysis was used to evaluate the performance of pre-processing. We first calculated the PCC value of log_2_-transformed ratio between any two samples from the same datasets. Then we estimated the correlation among samples of different platforms before and after pre-processing. The PCC values of any two samples should not show the dependencies on the platform after high-quality preprocessing.

### Profiling of chromosomal aberrations and pathway analysis

Based on the definition of gain and loss events, we conducted genome-wide profiling of CNAs for all the HCC samples included in the four datasets and calculated the frequencies of chromosomal aberrations for each sample. We then, using a Chi-square test, compared the frequencies of copy number gains and losses separately between HBV-HCC and HCV-HCC, or between HCC with and without viral infection. In further analyses, we only focused on those highly prevalent events of copy number gains and losses (frequency >25%). We first evaluated the correlation between any two CNAs by calculating the PCC value. Then, the genes in these segments with frequent gains/losses were mapped into the pathways derived from the Kyoto Encyclopedia of Genes and Genomes (KEGG) database [30]. The pathways that were significantly affected by the identified CNAs were determined by Fisher' exact test.

## Results

### Profile of chromosomal alterations in all HCC samples

**Figure 1:**
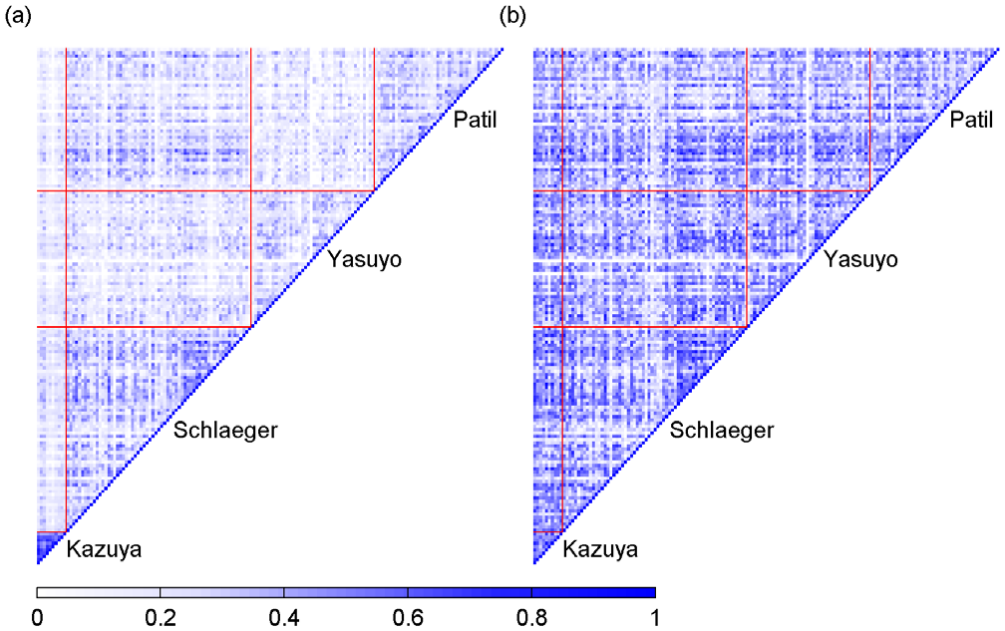
Comparison of correlations among log2-transformed ratios of 159 samples from four independent datasets before (a) and after preprocessing (b). In triangle (a) and (b), color points dotted in rectangles represent PCC values of log2-transformed ratio between any two samples from different datasets, while color points dotted in small triangles represent those from same dataset. The brightness of color blue is directly proportional to the value (0 – 1) of Pearson correlation coefficient.

**Figure 2:**
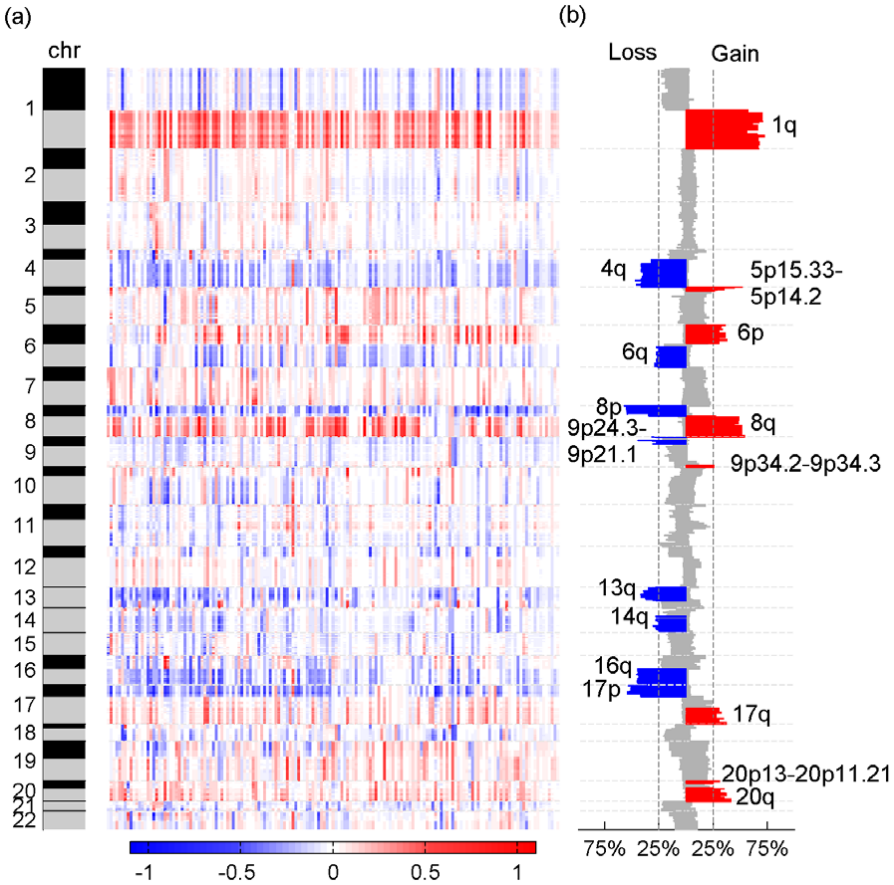
Profile of chromosomal alterations in all HCC samples (n = 159). **(a)**, heatmap of CNAs across all chromosomes. **(b)**, the frequencies of CNAs across all chromosomes. Copy number gain and loss events of chromosomal segments were determined using log2-transformed ratio thresholds of 0.141 and −0.136 derived from the k-means clustering analysis. The color brightness is directly proportional to the frequency of CNA. Color red represents copy number gain and color blue represents copy number loss. CNA: copy number alteration.

We profiled the CNAs (gains or losses) of chromosomal segments in human HCC through a meta-analysis of four available independent array CGH datasets including total 159 samples. To integrate all datasets from different platforms, the raw data were pre-processed and the pre-processing performance was evaluated using the PCC analysis. Our results indicated that the PCC values of individual samples within a platform were significantly higher than those between two different platforms before data pre-processing. After pre-processing, the correlation obtained for samples between different platforms after pre-processing was significantly improved with an increase of average correlation coefficient from 0.198 to 0.349 (Fig. 1). These data showed that the four independent datasets have been successfully transformed into a comparable form. Next, chromosomal gain and loss events were determined using log_2_-transformed ratio thresholds of 0.141 and -0.136 derived from the *k*-means clustering analysis and the frequencies of chromosomal aberrations were calculated for all HCC samples. Our results indicated that 85 chromosomal segments exhibited a frequent copy number gain (≥25%) and 88 exhibited a frequent copy number loss (≥25%). Detailed data was not presented here because of the space limitation. They are available when required. As shown in Fig. 2, the significant gains were mostly mapped to several broad chromosomal regions including 1q, 6p, 8q, 17q and 20q, as well as four narrow regions including 5p15.33- 5p14.2, 9q34.2-34.3, 20p13 and 20p11.21. Significant losses were frequently observed in 4q, 6q, 8p, 9p, 13q, 14q, 16q, and 17p. Among these regions, chromosome 1q was most significantly affected by copy number gain and chromosome 8p was most significantly affected by copy number loss. In particular, gains in 1q32.1, 1q24.1, and 1q21.3-23.3 were detected in >70% of all HCC samples, and losses in 8p23.3-21.3 were detected in >55% of the samples.

### Correlations between significant chromosomal aberrations

**Figure 3:**
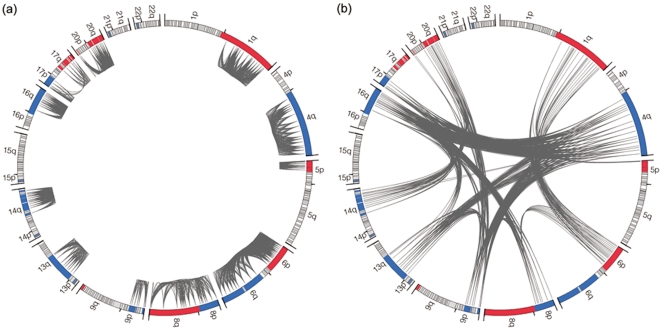
Correlations between significant chromosomal aberrations either located on the same (a) or different (b) chromosomes in all HCCs. Chromosomal segments with significant gain were highlighted in red and those with significant loss in blue. Spearman's rank correlation coefficient was calculated to assess the correlations between different chromosomal copy number gains and losses at the significant level of p<0.001.

Spearman's rank correlation coefficient was calculated to assess the correlations between different chromosomal copy number gains and losses described above at the significant level of p<0.001. Our results indicated that significant correlations existed between specific chromosomal aberrations either located on the same or different chromosomes and the significances from the same chromosome were usually higher than those from different chromosomes. As shown in Fig. 3(a), significant finding from the same chromosome were mainly identified for chromosomes 1q, 4q, 5p, 6p, 6q, 8p, 8q, 9p, 13q, 14q, 16q, 17p, 17q, 20p, 20q and between 6p and 6q, 8p and 8q, 17p and 17q, 20p and 20q. As indicated in Fig. 3(b), significant correlations mainly existed between 1q gains and 5p gains, and 6p gains or 4q losses, with a positive correlation coefficient of at least 0.25, 20q gains and 9p losses or 14q losses with a negative correlation coefficient of over −0.3. In addition, losses of 4q, 9p, 13q, 16q and 17p frequently co-occurred with a correlation coefficient ranging from 0.26 to 0.46. Significant correlations were also observed between 6p gains and 8p losses, 8p losses and 9p losses, and 14q losses and 16q losses.

### Canonical pathways significantly linked with chromosomal aberrations

To explore the potential effects of chromosomal aberrations implicated in the molecular mechanism of HCC development, we further analyzed the functional KEGG pathways enriched with genes located on the chromosomal segments with significant copy number alterations. Our results indicated that the genes affected by chromosomal aberrations were significantly enriched in 31 canonical pathways. The list of pathways together with the related gene information was summarized in Table 2. According to the biological function, these pathways were divided into three major categories: 18 immune-related pathways, 3 cancer-related pathways and 10 metabolism-related pathways. Among these pathways, antiviral immunity pathways were most significantly affected, such as the antigen processing and presentation pathway (hsa04612), RIG I like receptor signaling pathway (hsa04622), natural killer cell mediated cytotoxicity pathway (hsa04650), cytosolic DNA sensing pathway (hsa04623), toll like receptor signaling pathway (hsa04620), and cytokine and cytokine receptor interaction pathway (hsa04060). Moreover, we found many pathways were interrelated and driven by the presence of same or similar sets of genes. For example, seven immune-related pathways (hsa05320, hsa04612, hsa04622, hsa04650, hsa04623, hsa04620 and hsa04140) were driven by signals in the same core set of the interferon-alpha family genes (*IFNA1, IFNA10, IFNA13, IFNA14, IFNA16, IFNA17, IFNA2, IFNA21, IFNA4, IFNA5, IFNA6, IFNA7, IFNA8*) that are contiguously located within a 0.3 Mb region on chromosome 9p21.3. These genes play a vital role in the non-specific antiviral immunity that occurs at the early phase of viral infections. We also found that 10 immune-related pathways harbored another core set of genes, the human leukocyte antigen gene family [27]. In addition, six metabolism-related pathway (hsa00982, hsa00140, hsa00053, hsa00983, hsa00980, hsa00040) had the same core set of genes, the uridine diphosphate glycosyltransferase 2 family (*UGT2A1, UGT2A3, UGT2B4, UGT2B7, UGT2B10, UGT2B11, UGT2B15, UGT2B17, UGT2B28)*, that are contiguously located within a 2 Mb region on chromosome 4q13.2. Only a few cancer-related pathways were found to be significantly affected by the chromosomal aberrations identified in this study with a common gene set. However, several oncogenes and tumor suppressor genes are mapped to these pathways, such as *TP53, RB1,* and *MYC*, which have been shown to play an important role in hepatocarcinogenesis [21], [31].

**Table 2:** The functional KEGG pathways enriched with genes located on the chromosomal segments with significant CNAs in all HCCs.

Pathway	KEGG ID	Mapped gene number/Pathway gene number	*P* value
SYSTEMIC_LUPUS ERYTHEMATOSUS	hsa05322	94/140	<1.00E-13
AUTOIMMUNE THYROID DISEASE	hsa05320	38/53	1.57E-12
ANTIGEN PROCESSING AND PRESENTATION	hsa04612	48/89	1.52E-08
ALLOGRAFT REJECTION	hsa05330	23/38	2.78E-06
VIRAL MYOCARDITIS	hsa05416	37/73	3.85E-06
RIG I LIKE RECEPTOR SIGNALING PATHWAY	hsa04622	36/71	4.97E-06
NATURAL KILLER CELL MEDIATED CYTOTOXICITY	hsa04650	60/137	5.55E-06
ASTHMA	hsa05310	18/30	2.91E-05
CYTOSOLIC DNA SENSING PATHWAY	hsa04623	28/56	5.97E-05
TYPE I DIABETES MELLITUS	hsa04940	23/44	8.76E-05
LEISHMANIA INFECTION	hsa05140	33/72	1.58E-04
TOLL LIKE RECEPTOR SIGNALING PATHWAY	hsa04620	43/102	2.56E-04
GRAFT VERSUS HOST DISEASE	hsa05332	21/42	3.70E-04
REGULATION OF AUTOPHAGY	hsa04140	17/35	0.002
COMPLEMENT AND COAGULATION CASCADES	hsa04610	29/69	0.002
DRUG METABOLISM CYTOCHROME P450	hsa00982	29/72	0.004
STEROID HORMONE BIOSYNTHESIS	hsa00140	22/55	0.011
CYTOKINE CYTOKINE RECEPTOR INTERACTION	hsa04060	87/267	0.014
INTESTINAL IMMUNE NETWORK FOR IGA PRODUCTION	hsa04672	19/48	0.018
ASCORBATE AND ALDARATE METABOLISM	hsa00053	11/25	0.019
CELL ADHESION MOLECULES CAMS	hsa04514	46/134	0.021
NITROGEN METABOLISM	hsa00910	10/23	0.025
PROXIMAL TUBULE BICARBONATE RECLAMATION	hsa04964	10/23	0.025
SMALL CELL LUNG CANCER	hsa05222	30/84	0.027
AMYOTROPHIC LATERAL SCLEROSIS ALS	hsa05014	20/53	0.028
DRUG METABOLISM OTHER ENZYMES	hsa00983	19/51	0.036
METABOLISM OF XENOBIOTICS BY CYTOCHROME P450	hsa00980	25/70	0.037
BLADDER CANCER	hsa05219	16/42	0.037
RIBOFLAVIN METABOLISM	hsa00740	7/16	0.040
BIOSYNTHESIS OF UNSATURATED FATTY ACIDS	hsa01040	9/22	0.046
PENTOSE AND GLUCURONATE INTERCONVERSIONS	hsa00040	11/28	0.048

**Note:** The pathways that were significantly affected by the identfied CNAs were determined by Fisher' exact test.

KEGG, Kyoto Encyclopedia of Genes and Genomes; CNA, copy number alteration.

### Etiology-related chromosomal aberrations

**Figure 4:**
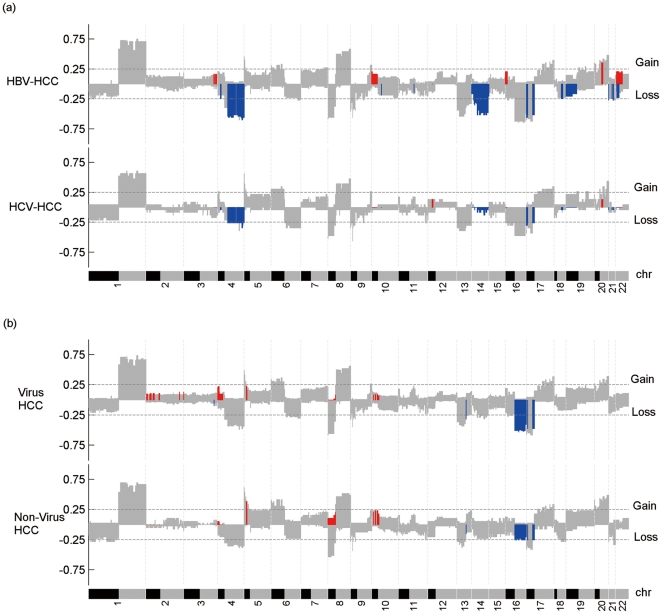
Profiles of etiology-related chromosomal aberrations in HCCs. **(a).** the comparison of chromosomal aberration profiles between HBV-related HCCs and HCV-related HCCs. **(b)**, the comparison of chromosomal aberration profiles between virus-related HCCs and non-virus-related HCCs. Copy number gains and losses with a significant difference in frequencies were highlighted in red and blue, respectively.

We investigated the profiles of etiology-related chromosomal aberrations in HCCs. As indicated in Fig. 4, HCC samples with different etiologies exhibited very similar profiles of chromosomal aberrations with only a few exceptions. When we compared the profiles of 48 HBV-related and 23 HCV-related HCCs, 14 copy number gains and 59 losses had significantly different frequencies. Almost all of these segmental gains and losses were harbored in HBV-related HCCs, except for gains of 12p13.1-12p12.3 and losses of 3q26.1-3q26.2 and 3q26.2-3q26.33 that appeared in HCV-related HCCs with significantly higher frequencies. The significant copy number gains and losses in HBV-related HCCs were most commonly located on chromosomal region 10p, and 14q and 4q, respectively. We also performed the comparison of chromosomal aberration profiles between virus-related HCCs and non-virus-related HCCs, and identified 27 gains and 17 losses showing a significant difference in frequencies. All significant losses were present in virus-related HCCs and most were located on chromosomal region 16q. In comparison, significant gains were mostly located on chromosomal regions 2p and 4p in virus-related HCCs, whereas chromosomal regions 8p and 10p in non-virus-related HCCs.

## Discussion

Meta-analysis of array CGH is valued as an integrated approach to simultaneously analyze information from different studies for the detection of chromosome aberrations with enhanced resolution and accuracy. A recent study has successfully performed a meta-analysis of array CGH data derived from a series of primary cancers to cluster tumor type by CNAs [24]. In this study, we, for the first time, conducted a cross-platform meta-analysis of HCC array CGH data obtained from different studies. We developed a preprocessing methodology to normalize the original data based on the MPCBS statistical algorithm and demonstrated that this data pre-processing exhibited an optimum performance on multi-platform integration.

In the present study, our meta-analysis identified 85 significant chromosomal gains and 88 losses. The majority of these significant aberrations were consistent with previous reports using conventional CGH approaches or including smaller sample size. In addition, our study identified several aberrations located in very narrow chromosomal regions, such as copy number gains on 5p15.33-5p14.2, 9q34.2-9q34.3 or 20p13-20p11.21 and copy number loss on 9p24.3-9p21.1 or 14q. The 5p15.33 regions contains *hTERT* gene, which encodes the catalytic subunit of telomerase, a critical component in telomere regulation. In an array CGH study, Yasuyo et al. [16] have reported that the gain of 5p15.33 was detected in 50% of HCC samples. Gain of this region has also been reported to be associated with progression of bladder cancer [32]. The 5p15.2 region hosts the potential target gene, delta catenin (*CTNND2*), over-expressed in prostate cancer [33]. The chromosomal region 9p24.3 harbors three putative tumor suppressor genes, *DMRT1*, *DMRT3* and *DOCK8*. A recent study has reported the frequent deletion of this region in squamous cell carcinoma of the lung [34]. Furthermore, our results indicated that significant correlations existed between chromosomal aberrations either located on the same chromosome or the different chromosomes, suggesting that these aberrations might appear non-randomly. It remains to be further elucidated whether there are sequential correlations among the occurrence of these genomic aberrations in the development and progression of HCC.

We also compared genomic aberrations between HCCs with different etiologies and observed two noteworthy findings. First, our study found that HCCs with different etiologies exhibited very similar profiles of chromosomal aberrations. Second, we found that chromosomal aberrations appeared more frequently in HBV-HCCs than in HCV-HCCs, and in virus-related HCCs than in non-virus-related HCCs. These findings suggest that virus infection, especially HBV infection, may play a major role in hepatocarcinogenesis by causing chromosomal aberrations. In our analyses, we observed that the significant copy number gains were most commonly located on chromosomal region 10p and significant losses on 14q and 4q in HBV-related HCCs. Yeh et al. have reported that allelic loss at chromosome 4q21-23 occurs frequently in human hepatocellular carcinoma [35]. Fas-associated phosphatase-1 (*FAP-1*) gene, a potential candidate TSG, is located in this region. Another study has demonstrated that three regions at 14q exhibit the frequent loss of heterozygosity in head and neck squamous cell carcinoma. Several candidate TSGs such as *CHES1*, *BMP4*, *SAV*, and *PNN* exist in these region [36]. These TSGs may play critical roles in hepatocarcinogenesis. Previous CGH studies have also analyzed the CNA profiles of HCCs with HBV or HCV infection. One study has reported that the amplification of 11q13, which corresponds to the chromosomal region harboring the genes for cyclin D1 and hst-1, is frequently observed in HBV-positive HCCs. In contrast, loss of 10q has been detected exclusively in HCV-positive HCCs [14]. Another study has demonstrated that a single high level amplification is only seen on 5q21 in HBV-related HCCs [37]. In addition, Christof Schlaeger et al compared HBV- and HCV-associated HCCs by array CGH, and observed that major differences in the frequencies of gains were at 1q32.1, 7q22.1, 10q26.3-qter in HCV- positive tumors, and losses at 4q34.3-qter, 9p24.3, and 14q21.2-32.33 were more significant in HBV–positive tumors [27]. The inconsistency among these results is possibly due to the different technology platforms and sample selection. Further validation using large sample size is warranted.

The tumorigenesis of HCC is a multi-step and multi-factorial process. Many attempts have been made to identify the abnormal pathways underlying hepatocarcinogenesis. Epidermal growth factor (EGF) signaling pathway is one of the most thoroughly evaluated signaling cascades in human HCC [38]. Genetic evidence has been also provided by a recent study showing gains of chromosome 7 in human HCC, where *EGFR* is located. A series of studies have also found the dysregulation of pathways involved in cellular differentiation and proliferation in HCC, such as the WNT canonical pathway and the Hedgehog signaling pathway (HH) [39], [40], [41], [42]. Interestingly, the most noteworthy finding of this study is that the genes affected by chromosomal aberrations in HCC were most significantly enriched in antiviral immunity pathways, followed by the cancer-related pathways and metabolism-related pathways. This is highly biologically plausible, given the important functions of immunity pathways in the response to viral infection and chronic inflammation [43]. Previous studies have provided various lines of experimental evidence in accordance with our findings. For example, Kirsten et al. have reported a significant amplification of 6p21.3 that corresponds to the HLA-region in ovarian cancer [44]. In addition, somatic deletions in chromosomes 9 and 22 have been studied in 21 paired HCC and adjacent tumor-free liver tissue samples [45]. Among informative HCC cases, the highest rates of loss of heterozygosis (LOH) were observed for 9p21 (40% or 4/10 at IFNA) and 9q23 (23% or 3/13 at D9S318). Clinical data indicate that chromosome 9p21 deletions occurred preferentially in larger tumors (>5 cm diameter). Another study has also reported that LOH on IFN-alpha locus at 9p21 is a prognostic indicator of bacillus Calmette-Guerin response for non-muscle invasive bladder cancer [46]. Similarly, Roman et al. [47] has investigated the chromosomal aberrations in head and neck squamous cell carcinomas by array CGH and found that the genes involved in cytokine-cytokine receptor interaction pathway were the largest group of genes (23/149) showing chromosomal gains. The copy number of several interleukin genes (*IL10, IL19, IL20* and *IL24*) mapped to1q32 have been significantly increased and all these genes were previously found to be associated with the development of HNSCCs. In addition to the interleukins at 1q32, there were amplified genes from other regions of the genome, such as *CCL26, EGFR, IL17R, IL28A, IL29, IL2RB, IL12RB1, VEGF, XCL2, TNFSF9* and *CD40*. Taken together, these lines of evidence strongly suggest that the functional aberration of antiviral immunity pathways may be significantly involved in the development and progression of HCC.

In summary, we conducted a cross-platform meta-analysis of HCC array CGH data. We identified multiple chromosome regions with significant aberrations in genes enriched in antiviral immunity pathways. Our results provide a sketchy description of the genomic alterations in HCCs and generate further testable hypotheses that the deficiency of antiviral immunity pathways may play key roles in hepatocarcinogenesis.
